# Tobacco endgame measures and their adaptation in selected European countries: A narrative review synthesis

**DOI:** 10.18332/tpc/186402

**Published:** 2024-04-18

**Authors:** Otto Ruokolainen, Hanna Ollila, Tiina Laatikainen, Salla-Maaria Pätsi, Giulia Carreras, Giuseppe Gorini, Dolors Carnicer-Pont, Zsuzsa Cselkó, Romain Guignard, Maria Karekla, Biljana Kilibarda, Helena Koprivnikar, Angeliki Lambrou, Viêt Nguyen-Thanh, Efstathios Papachristou, Sotiria Schoretsaniti, Milena Vasic

**Affiliations:** 1 Department of Public Health and Welfare, Finnish Institute for Health and Welfare, Helsinki, Finland; 2 Institute of Public Health and Clinical Nutrition, University of Eastern Finland, Kuopio, Finland; 3 Institute for Cancer Research, Prevention and Clinical Network, Florence, Italy; 4 Grupo de Investigación en Control del Tabaco, Institut d'Investigació Biomèdica de Bellvitge, L'Hospitalet de Llobregat, Barcelona, Spain; 5 Centro de Investigación Biomédica en Red de Enfermedades Respiratorias, Madrid, Spain; 6 Programa de Prevenció i Control del Càncer, Institut Català d'Oncologia, L'Hospitalet de Llobregat, Barcelona, Spain; 7 National Korányi Institute of Pulmonology, Budapest, Hungary; 8 Prevention and Health Promotion Department, Santé publique France, Saint-Maurice, France; 9 University of Cyprus, Nicosia, Cyprus; 10 Institute of Public Health of Serbia ‘Dr Milan Jovanovic Batut’, Belgrade, Serbia; 11 National Institute of Public Health, Ljubljana, Slovenia; 12 Directorate of Epidemiology and Prevention of Non-Communicable Diseases and Injuries, National Public Health Organization, Athens, Greece

**Keywords:** tobacco, review, smoking, policy, tobacco control, Endgame

## Abstract

Due to the continued detrimental effects of tobacco use, a growing number of countries are embracing the idea of tobacco endgame, meaning ending the tobacco epidemic instead of controlling it. This narrative review aims to synthesize and update the evidence from earlier scientific reviews on effective tobacco endgame measures, as well as to assess their integration to current national strategies among European countries with official tobacco endgame goals. The synthesis of the prior scientific literature found most evidence on product-focused and some evidence for supply-focused policies. Little evidence was detected for user- and institutional-focused measures. An update for the tobacco-free generation measure showed uncertainty in reducing smoking prevalence, especially for adolescents’ reactions to age-restrictive laws. All the countries that established a tobacco endgame strategy have included product standards in their measures, predominantly based on European Union regulations on conventional tobacco products, yet standards above this level and considering other products were also common. Cessation measures were given strong emphasis in strategies, yet none of the countries linked these to specific endgame measures. Despite commonly mentioning vulnerable groups, such as youth and pregnant women, adoption of measures to reduce tobacco use among these groups was scarce. Lastly, the decline in tobacco use seems to be modest, implying challenges in meeting the endgame goals. To meet these goals, European countries should reinforce the implementation of known effective tobacco control measures such as tax increases. Furthermore, new innovative strategies and measures to meet the objective of an endgame should be explored.

## INTRODUCTION

Since 2005, key global tobacco control regulations have been harmonized through the entry into force of the WHO Framework Convention on Tobacco Control (WHO FCTC). While most focus has been on the implementation of the measures required and recommended in the WHO FCTC, recently, more attention has been given to Article 2.1, which encourages countries to implement measures beyond the treaty to provide ultimate protection of health. This aligns with the paradigm shift where countries are embracing the idea of tobacco endgame – meaning ending the tobacco epidemic instead of reducing and controlling it^1^.

Tobacco endgame is defined as aiming for a minimal level of tobacco use in the general population – preferably with a measurable goal in a clearly defined time frame^1^. Following the developments at the national level, a ‘Tobacco-Free Generation’ (TFG) goal was set for the EU member states in 2021 in Europe’s Beating Cancer Plan^2^. This has been defined in practice as reaching <5% tobacco use prevalence by 2040 at the EU level. As the prevalence of smoking among adults in the EU was 23% in 2020^3^, reaching the target likely requires both stronger implementation of key regulations – WHO FCTC including the MPOWER ‘best buys’ – as well as implementation of innovative measures to reach tobacco endgame.

In the implementation roadmap of the EU Cancer Plan^4^, the TFG goal is addressed through foreseen revisions of key EU tobacco directives for product regulation, marketing, and taxation. Furthermore, the EU-funded Joint Action on Tobacco Control 2 (JATC2) project is expected to facilitate the implementation of tobacco control measures through enhanced collaboration between the European countries^5^. Work package 9 (WP9) of JATC2 specifically addresses the best practices to develop effective and comprehensive tobacco endgame strategies. This is done by identifying and assessing forward-looking tobacco control policies and tobacco endgame strategies for the European region, also considering the integration of cessation support to these, and exploring and promoting best practices in their development, implementation, and evaluation. The final deliverable of this WP is an online toolkit to support awareness raising and national actions, available through the www.jaotc.eu-website as of June 2024^6^.

The so-called harm reduction or harm modification measures, often promoted by the tobacco industry as alternatives for smoking^7^, such as the use of electronic cigarettes or heated tobacco products, have not been considered in WP9 as tobacco endgame measures should aim at ending the use of tobacco and other nicotine products.

The research questions and their sub-questions of this narrative review are as follows:

1. What is the current knowledge on the effectiveness of proposed tobacco endgame measures, and what are the research needs?
2. Among European countries with official tobacco endgame goals, how has ‘endgame’ been operationalized in their national strategies? How has the tobacco endgame been defined in these countries? Have tobacco endgame measures been included in the strategies? Has support for tobacco cessation been integrated into the strategies? Have vulnerable population groups been considered in the development, implementation, and evaluation of tobacco endgame goals? Is the progress toward the endgame goal evaluated? Are the countries progressing toward the endgame goal with the selected measures?

To facilitate the understanding of tobacco endgame strategies and the development of national goals, this narrative review aims, first, to synthesize and update the findings of earlier scientific reviews on the evidence of effective tobacco endgame measures and research needs. Second, it seeks to investigate the current situation in Europe, focusing on the content of the endgame strategies and adaptation of different measures in countries that have set an endgame goal.

A narrative review of the adopted endgame policies in European countries was conducted. A narrative review can include variety of studies with the aim of providing an overall summary, including interpretation and critique, of a certain topic^8^.

## DEVELOPMENTS

### Synthesis of existing literature on the current knowledge on the effectiveness of proposed tobacco endgame measures and the research needs

The most recent comprehensive review on tobacco endgame by Puljević et al.^9^, was an update of a previous one by McDaniel et al.^1^ and included 49 publications through a search on five databases (PubMed, CINHAL, SCOPUS, Web of Science, Embase) and the inclusion of additional reports from a Google search and expert opinion^9^. This review described endgame policies and identified endgame gaps and research priorities.

Since the Puljević et al.^9^ review refers to recently published articles (search up to 2021), an update was carried out only for feasible policies (i.e. TFG) for which there were evidence gaps, by applying the same search string in PubMed used by Puljević et al.^9^ to an updated timeframe (see Supplementary file Material A for the search string). A policy was defined as feasible in Europe on the basis of an expert opinion in JATC2 WP9 on its possible advocacy and social acceptability. The literature on each policy measure was considered sufficient if more than five articles were identified on the topic.

### Inclusion criteria for the European endgame countries

The second aim of this narrative review was to identify countries that adopted a tobacco endgame goal in Europe. An endgame country refers to a country that has defined an endgame goal aiming at a minimal level or no tobacco use in the population in an official governmental document. The focus was outlined on European countries, which corresponded with the definition of the target group of the JATC2. The initial search of governmental strategy documents and other publications, such as news pieces, on regulatory changes, indicating the existence of a tobacco endgame goal was conducted between August 2022 and January 2023 without restrictions on document publication dates. The search was appended during the writing of this study. The search was first done by one author and later confirmed by three other authors. Based on the search, ten countries were included in the analyses: Belgium, Finland, France, Ireland, the Netherlands, Norway, Slovenia, Sweden, and as part of the United Kingdom, England and Scotland. Most of the included policy documents were national, long-term tobacco control strategies; some of them included action plans or roadmaps, while some were broader public health strategies (the Netherlands, Norway) or integrated strategies of substance use/addiction policies (Sweden). The authority responsible for the enforcement of the strategy was usually the Ministry/Department of Health, whereas some strategies described the responsibilities of other, mostly governmental organizations/departments (Belgium, Ireland, Sweden). In the Netherlands, the strategy enforcement was appointed by signing a multisectoral agreement including, for example, civil society and business communities, municipalities, and healthcare, welfare, and education sectors. While several policy documents included descriptions of funding (either currently effective or possibilities for future funding), fewer details of its allocation and sufficiency for the enforcement of the strategies were described.

The data on the existence of tobacco endgame goals and strategies were complemented as needed by information provided in the answers gathered with the JATC2 WP9 questionnaire on tobacco endgame strategies. The questionnaire was distributed to all the WHO FCTC focal points in the WHO European region from 15 September 2022 until 13 January 2023. The questionnaire is available as part of the project indicator compendium^6^.

### Measures

Policies that have been identified to have the potential to achieve a tobacco endgame were selected and grouped into product, user, market/supply, and institutional structure-oriented tobacco control measures based on two earlier endgame reviews and syntheses^1,9^. In the JATC2 WP9, one task was to assess the integration of tobacco cessation support to tobacco endgame strategies. Therefore, it was also assessed whether smoking cessation measures were included in the strategy and whether they related to specific endgame measures.

After identifying the countries, a further search of policy documents, governmental websites, scientific articles, and media coverage, such as news, was conducted to assess what measures the countries had adopted to achieve the tobacco endgame goal. Texts in the local language were used with the help of digital translator services, as needed. WP9 partners in each country did the final check of the gathered information and the translation to ensure factuality. In the case of identification of previous endgame policy documents for a country, only the latest one was taken into consideration. However, in some cases (such as in England), the policy documents overlapped, yet were not mutually exclusive. In these cases, more than one policy document was reviewed. The country policy documents included are listed in Supplementary file Material B.

*Evaluation plan and vulnerable populations*

The countries were considered to have an evaluation plan if the strategy documents included concrete actions on how to evaluate or monitor the strategy implementation. The documents were also examined to determine whether vulnerable populations (e.g. children and people with low socio-economic positions) were considered in the strategy, for example, by mentioning how tobacco use affects different population groups. It was also assessed whether vulnerable populations are considered in the implementation of the strategy.

*Prevalence of use*

To get a comprehensive picture of the level of product use in the included countries, four authors selected the indicators that were utilized to describe smoking, tobacco, e-cigarette, and smokeless tobacco use. Indicators reported by the WHO were selected because of the comparability of the data.

### A synthesis of existing scientific reviews on the effectiveness of proposed endgame measures

Tobacco endgame policies were grouped into four broad categories as aforementioned: product-focused, user-focused, market/supply-focused, and institutional structure-focused (Table 1)^1,9^. Based on aspects of policy implementation, the authors identified policies that reached an evidence synthesis, i.e. for which results from multiple empirical studies were identified, selected, and combined to draw conclusions^10^. Evidence synthesis was considered as an indicator of progression toward the translation of research evidence into policy^9^ (Table 1).

**Table 1:** Synthesis of existing scientific review^9^ on endgame measures and update on tobacco-free generation policies

*Policy category*	*Policy description*	*Sufficient literature^a^*	*Main evidence*	*Main evidence gaps*
**Product-focused**	1. Mandate very low nicotine content (VLNC) for smoked tobacco products to make them non-addictive or less addictive.	Yes (26)	Effect on notable reduction in cigarette smoking, smoking prevalence and related harm. Public support for VLNC standard. Impact on the use of other nicotine products or drugs. Impact on people experiencing mental illness, socio-economic disadvantage, pregnancy.	Feasibility. Impact of the policy in terms of mental and physical health outcomes, use of alternative nicotine products, other substance use, and priority populations. Tobacco industry responses. Potential effect on the illicit market. Impact of public communication and education strategies to maximize policy benefits. Nicotine threshold for addiction.
	2. Set product standards for nicotine products that make combustible tobacco products unappealing or removed from the market for exceeding toxicity thresholds.	No (1)	Evidence on public support for the policy for banning menthol.	Feasibility. Tobacco industry responses, e.g. substituting banned constituents with other harmful ingredients.
**User-focused**	3. Require consumers to obtain a purchaser’s licence or medical prescription to purchase tobacco.	No (0)		
	4. Restrict tobacco sales by year born (tobacco-free generation).	No (4)	Modelling population health impact of tobacco-free generation. Key legal and ethical issues of the tobacco-free generation. The implementation of this policy alone in a simulation model is unlikely to achieve a 5% smoking prevalence in 10 years. If combined with policies of denicotinization and retail outlet reduction, this policy could have major impacts on reducing inequities in health. Indirect evidence on youth defiance; universal laws may be better perceived by adolescents; age-specific laws are perceived as a form of youth control.	Policy effectiveness Exact meaning of human rights articles within the sphere of public health.
**Market/supply-focused**	5. End commercial retail sale of combustible tobacco (abolition).	No (2)	Varying public support (12%–88%).	Empirical evidence on effectiveness in achieving tobacco endgame.
	6. Set a regularly reducing quota on the volume of tobacco products manufactured or imported into a country (‘sinking lid’).	No (2)	Simulation of implementation in New Zealand. Simulated impact on health gain and cost saving.	Policy effectiveness, practicality or legality. Substitution relationships between different tobacco products.
	7. Actions that reduce the commercial viability of tobacco companies, such as a ‘corporate death penalty’, or criminal charges, requiring compensation for full impacts of tobacco use, or limiting profitability.	No (0)		
	8. Increases in tobacco tax that make tobacco products generally unaffordable.	Yes (7)	Effect on health improvement and on decrease of smoking prevalence. Decreased health system costs.	Country-specific research on price elasticity variation by age and social groups. Impact of tax increase in conjunction with other policies.
	9. Restrictions on tobacco retailer density/location/type/licensing that substantially reduce tobacco availability.	Yes (10)	Effective for reducing population-level tobacco use and health system costs.	Feasibility in some European countries, e.g. France, Italy and Spain, where tobacconists are exclusive tobacco retailers.
**Institutional structure-focused**	10. Transfer management of tobacco supply to an agency with a mandate to phase out tobacco sales.	No (0)		
	11. Performance-based regulation whereby tobacco companies are required to meet smoking prevalence targets or be fined; or manufacturers pay a levy based on sales volume similar to ‘polluter pays’ schemes.	No (0)		

a Sufficient studies: n>5.

The tobacco endgame policy with the most evidence was the product-focused policy on mandatory very low nicotine content (VLNC) standard, with 26 studies that addressed various aspects of the topic (policy 1, Table 1). The main goal of the policy is to create less-addictive products with the aim of reducing tobacco use. Although a VLNC standard is yet to be implemented in any country, the New Zealand government announced in 2021 that it will implement such a measure by 2025 (yet this and other tobacco endgame measures have been since repealed when the government changed) and the US FDA has also proposed such a measure. Despite VLNC being the most studied policy, there are still several evidence gaps. First, the feasibility of VLNC is unknown. Moreover, there is no evidence of its potential effects in terms of mental and physical health outcomes, possible transition to other tobacco or nicotine products or other substance use, and priority populations. Then, industry and illicit market responses are unknown. Finally, the threshold for developing dependence is currently unknown^9^.

Another product-focused policy is setting product standards that would make combustible tobacco products unappealing, such as raising the pH of cigarettes or banning menthol (policy 2, Table 1). This policy was studied in a narrative review, which presented various proposals to redesign cigarettes but reported evidence only on public support for the policy for banning menthol^1^.

Less evidence was identified for market-/supply-focused policies. Increasing tobacco taxes, making tobacco products generally unaffordable (policy 8, Table 1), was analyzed in seven studies. The policy was proven to significantly reduce smoking. The main gap that makes this policy unfeasible is that increases in tax levels necessary to achieve endgame goals could be politically difficult to implement in a short time frame. However, the Australian Government succeeded in increasing tobacco prices from around 4 euros per 20 cigarette package in 2001 to around 24 euros in 2021^11^. Ending sales of tobacco products (implemented in two local USA government areas) were analyzed in two studies, which presented large gaps in its effectiveness in achieving endgame goals and with no feasibility studies conducted (policy 5, Table 1). Also, the so-called ‘sinking-lid’ policy, i.e. setting a regularly reducing quota on the volume of tobacco products manufactured or imported into a country (policy 6, Table 1), was analyzed in two simulation studies that explored its practical implementation in New Zealand and simulated gains in health and cost savings^12,13^.

Ten studies considered a policy of restricting tobacco retailing as part of an endgame strategy, with the aim to reduce both adolescent and adult smoking rates (policy 9, Table 1). This policy was implemented in Hungary, where the number of tobacco-selling shops was reduced from nearly forty thousand to a few thousand, and set as a government policy goal in Australia. However, there might be significant differences between countries in Europe; for example, in Italy, France, and Spain, selling tobacco is allowed only for tobacco-specific retailers, and opposition from the retail sector will not be negligible when adopting such a policy^14,15^.

No evidence was found for several policy categories: the user-focused policy on requiring consumers to obtain a purchaser’s license or medical prescription to purchase tobacco (policy 3, Table 1); the supply-focused policy on actions that reduce the commercial viability of tobacco companies (policy 7, Table 1); and the institutional structured-focused policies on transferring management of tobacco supply to an agency with a mandate to phase out tobacco sales (policy 10, Table 1). Similarly, no evidence was found on performance-based regulation whereby tobacco companies are required to work to reduce smoking prevalence (policy 11, Table 1).

Finally, the user-focused policy for TFG (policy 4, Table 1) was considered in four studies reporting substantial population-level health improvements, even if the potential of achieving the endgame goal of minimal smoking prevalence is not necessarily achieved if implemented alone. A recent simulation study in New Zealand considered a combined package of denicotinization of retail tobacco, a 95% reduction in retail outlets, and TGF. The authors estimated it was associated with a large smoking prevalence reduction^16^. The policy was implemented in Balanga City Council (Philippines) in 2016 by banning the sale and use of all tobacco products for those born on or after 1 January 2000, thereby becoming the first in the world to embody the TFG idea^17^. Moreover, unsuccessful attempts at implementation were carried out in New Zealand using 2009 as the cut-off date^18^ and in Australia, in the state of Tasmania^19^. In Tasmania, the policy was introduced into parliament, but it lapsed when parliament was prorogued in 2018, despite the high public support for this proposed legislation (75% among Tasmanian adults and 72% among current smokers)^20^. In 2021, the policy was adopted in Brookline City Council (USA), using again the 2000 cut-off^21^. A European Citizens’ Initiative was presented in August 2022 to achieve a tobacco-free environment and the first European TFG by 2030 by advocating for ending the sale of tobacco and nicotine products to citizens born after 2010^22^.

*An update of review on the policy regarding tobacco-free generation (TFG)*

The update of scientific reviews specific to the TFG policy resulted in nine articles, two of which were already included in the 2022 scoping review^9^, and five that did not include substantive evidence synthesis^23-27^. Two studies thus resulted from the update^16,28^.

Berrick^28^ collected indirect evidence on the relevance and implications of adolescent psychology for minimum-age laws. This report highlighted the uncertain efficacy of age-restrictive tobacco laws in reducing adolescent smoking prevalence, recalling a recent review^29^ that noted the absence of studies evaluating the effects of an age-of-sale ban as distinct from other, simultaneously enacted policies. Additionally, uncertainty around minimum-age laws was due to the fact that they are advocated by tobacco industries that are well aware of adolescent psychology and their reaction to age-restricted laws. This is exemplified by three aspects. First, being that tobacco use is legal and thus safe for adults, then presumably, the real aim of the law could be considered as youth control rather than tobacco control. Second, reactance theory predicts that youth who are legally excluded from the product will find it more desirable. Finally, in the presence of underage laws, cigarettes become a symbol of the onset of maturity. Another aspect of indirect evidence about adolescent reactions to age-restrictive laws was given for laws concerning motorcycle helmets, with youth defiance of an age-restricted helmet law that disappeared when replaced by a universal law^30^. This supports studies of youths’ reactions, which highlight that universal laws may be perceived by adolescents as intended for protective benefit, whereas age-specific laws signal authorities’ desire for youth control. However, raising the age of sale to 21 years seems to have a positive impact on reducing smoking; Tobacco 21 laws reduced smoking rates of youths aged 18–20 years by 2.5 to 4 percentage points^31-34^.

The second study resulting from the review update is a simulation model with a hypothesized effect of reducing smoking initiation by 90% in 10 years from the implementation of the TFG in 2022 in New Zealand^16^. The study showed that a <5% smoking prevalence will not be achieved in 2040 by sex and ethnic group. If combined with VLNC policies and retail outlet reduction, the TFG policy would achieve a rapid reduction in smoking prevalence, mainly due to VLNC, and would reduce health inequity between Māori and non-Māori^16^.

Summarizing, studies resulting from the review update did not report evidence to support the law for reasons related to adolescent psychology and the limited effectiveness of the law in reducing prevalence if implemented alone.

*Research needs*

Further research is needed to improve estimates of the effectiveness of endgame policies that are small or poor for most interventions due to the lack of implementation. The quantitative examination of examples of implementation (even partial) would be useful^9^. Moreover, further studies are needed to evaluate the impact of endgame policies in vulnerable populations and in different countries and regions, such as low-income countries. These populations have notably high levels of smoking prevalence, being in a previous stage of the tobacco epidemic^35^ compared to non-priority or high-income populations. There is little research on the feasibility and effectiveness of implementing endgame strategies at an early stage of the tobacco epidemic^9^.

### National tobacco endgame definitions

Results considering endgame definitions and measures were obtained from the assessment of the policy documents, supplemented with WHO FCTC focal point questionnaire^6^ if needed. There were different definitions of the selected endgame goals. Most countries have defined it by the prevalence of tobacco (or tobacco and nicotine) product use (Table 2). Other definitions were related to TFG or tobacco-free society (TFS). Some of the countries incorporated both prevalence and TFG or TFS definitions in their endgame objective (the Netherlands, Norway, England). Most of the countries included combustible tobacco in their endgame goals, while some countries, such as Finland, Norway, and Slovenia, also included other tobacco or nicotine products (excluding nicotine replacement therapy), such as electronic cigarettes, in their endgame goals.

**Table 2:** Definitions of the current endgame goals of the European endgame countries

*Country*	*Definition*	*Year launched*	*Endgame goal year*
**Belgium**	<5% of population aged ≥15 years use tobacco daily by 2040	2022	2040
**Finland**	Nicotine-free Finland by 2030 End the use of tobacco and other nicotine-containing products by 2030 <5% of the adult population use tobacco and nicotine products daily	2016 (first in 2010)	2030 (first by 2040)
**France**	Children born since 2014 become the first non-smoking generation of adults by 2032 A generation in which 95% of people do not smoke (<5% smokers)	2018	2032
**Ireland**	Tobacco-free Ireland by 2025 <5% smoking prevalence rate of the Irish population	2013	2025
**The Netherlands**	In 2040 <5% of the residents of the Netherlands aged ≥18 years will smoke 0% of young people (smoke-free generation) and pregnant women will smoke	2019	2040
**Norway**	Tobacco-free generation 2010. Children born in 2010 and later will not use tobacco and nicotine products The proportion of daily smokers and daily users of snus must be <5%	2023 (first in 2013)	Not defined
**Slovenia**	Tobacco-free society by 2040 <5% of the population aged ≥15 years use tobacco and other nicotine-containing products	2022	2040
**Sweden**	<5% smoking prevalence by 2025	2016	2025
**United Kingdom**	England Smoke-free by 2030 ≤5% smoking rate a smoke-free generation	2019	2030
	Scotland ≤5% smoking prevalence of adults by 2034 a tobacco-free generation (children born in 2013 by the age of 21 years)	2013	2034

### Have tobacco endgame measures been included in the strategies? Has support for tobacco cessation been integrated into strategies?

*Tobacco endgame measures*

All of the countries included some product standards in their endgame measures, which were commonly based on EU Tobacco Products Directive (TPD)^36^ regulations and mainly referred to bans on characterizing flavors and additives. Some countries (Belgium, Finland, Norway, and Slovenia) also extended flavor bans to products other than cigarettes and roll-your-own tobacco regulated by the TPD. Plain packaging was adopted in all the countries except Sweden. Norway included warnings on individual cigarette sticks, and Scotland included pack inserts in their strategies. France was the only country that acknowledged the reduction of nicotine levels in cigarettes. England and Scotland included the possibility of restricting tobacco possibility of restricting tobacco sales by year born in their strategies, yet none of the countries, yet none of the countries included a license or prescription to purchase cigarettes in the strategies. Considering institutional structure-focused measures, none of the countries included transferring management of tobacco supply to an agency or required tobacco companies to meet smoking prevalence targets defined by the governmental tobacco control policy.

Market/supply-focused measures were infrequently included in the strategies. None of the country’s strategies included measures to end the commercial retail sale of combustible tobacco, to reduce the quota of tobacco products manufactured or imported into the country, or to reduce the commercial viability of tobacco companies. Additionally, none of the strategies included tax increases to make tobacco products generally unaffordable, although conventional tax increases were commonly included (yet they did not specify the type of tax: ad valorem or specific excise tax). Some countries adopted taxation on new products such as e-cigarettes or snus (for example, Finland, Slovenia, and Sweden). Strategies of all the ten countries included measures to reduce tobacco availability, for example, by licensing or registry systems and banning sales in certain places (near schools, festivals, hospitals, etc.) or banning distance sales. For example, in the Netherlands, tobacco sales are going to be restricted gradually only to tobacco shops, which is already in place in France.

*Integration of tobacco cessation support*

All the countries included a number of different smoking cessation support measures in their strategies. Cessation support measures included, for example, increasing and developing the services, increasing the availability and affordability of cessation medicines (for example, in France, by reimbursing nicotine replacement therapy), and preparation (Ireland, Slovenia) or enhancing the implementation (Finland) of national care guidelines. Even though cessation measures were given strong emphasis in the strategies, none of the countries linked cessation measures to specific endgame measures.

### Have vulnerable population groups been considered in the development, implementation, and evaluation of tobacco endgame goals?

All the countries at least mentioned vulnerable groups in their strategies, usually children, pregnant women, people experiencing mental illness, and smokers with low socio-economic position (Table 3). This was especially emphasized in Sweden, which also was, in addition to England, the only country considering people according to sexual orientation in their strategies. Even though vulnerable groups were commonly mentioned, only a few countries planned exact measures outside of smoking cessation support measures for these groups. In Scotland and France, there are explicit objectives for the prevalence reduction of smoking in groups with low socio-economic position. In Belgium, mentions about vulnerable groups concentrated on measures of smoking cessation in different settings, such as healthcare and social care, educational institutions/schools, and workplaces. In Sweden, supervision of tobacco and nicotine products and prevention work in schools were emphasized.

**Table 3:** Included vulnerable groups and related actions in the current strategies among European tobacco endgame countries

*Country*	*Vulnerable population groups are included in the strategy*	*Examples of actions on vulnerable groups (either general or specific groups)*
**Belgium**	Minors, social groups consuming more tobacco, patients with psychiatric disorders	General: close co-operation with health care and social care to ensure proximity to the most vulnerable groups, educational support in quitting assistance in different sites (school, work, healthcare, local government, leisure). Multicultural origin: setting up smoking cessation centers in hospitals and strengthening the link between tobacco specialist and hospitals. Youth: smoke-free environments.
**Finland**	Youth, pregnancy, mental health patients, low socio-economic position, unemployed	General: supporting smoking cessation in groups where smoking is common. Youth: raising minimum age, sport club tobacco and nicotine use prevention, tobacco-free playgrounds and beaches.
**France**	Children/youth, pregnancy, low socio-economic position, low income, unemployment, incarcerated people, mental health patients	General: strengthen the support for smoking cessation, strengthen the accessibility of nicotine replacement therapies; ‘Tobacco-free Healthcare Facilities’ including mental health facilities and maternity wards, smoke-free environments for incarcerated people. Children: reduce the attractiveness and availability of tobacco and nicotine products, facilitate support for smoking cessation (especially in vocational schools), promote non-smoking areas around entrance areas of education establishments.
**Ireland**	Children, young adults, retired, low socio-economic position, pregnancy	General: smoking cessation staff trained to deal with specific groups. Children: smoke-free environment (schools, child care), prohibit sale in events for those aged <18 years, campaigns.
**The Netherlands**	Children/youth, pregnancy, low socio-economic position	Children: smoke-free environment (schools, zoos, sports clubs). Pregnancy: smoke-free-pregnancy campaign, cessation training courses.
**Norway**	Children/youth, pregnancy, immigrant groups, low socio-economic position	General: national tobacco cessation program to reduce inequalities in health.
**Slovenia**	Children/youth, pregnancy, hospitalized/mental health patients, low socio-economic position	General: equal access to programs on prevention of initiation and cessation regardless of age, gender and socio-economic position, education and geography, campaigns on smoking cessation aimed at vulnerable groups, extension of smoke/aerosol-free places. Children: school programs.
**Sweden**	Children, socio-economic groups, ethnic background, age, gender, sexual orientation	Minors: supervision of tobacco and nicotine products, prevention work in schools.
**UK: England**	Youth, pregnancy, mental health, low socio-economic position (income, occupation), ethnicity, incarcerated people	Young people: prohibiting selling tobacco products for anyone born on or after 1 January 2009, restricting the flavors and description and the sale of vapes, point-of-sale display regulation for vapes, regulating vape packaging. Pregnancy: brochures and behavioral support for pregnant smokers to stop smoking, guidance on how to help quit, CO testing. Mental health: materials for staff, gather evidence how to reduce prevalence and integrate services.
**UK: Scotland**	Young people, pregnancy, low socio-economic position, incarcerated people, mental health patients	Young people: raising the age of sale, marketing campaigns. Prison: integrated smoking cessation services. Pregnancy: support for smoking cessation.

### Is the progress toward the endgame goal evaluated?

All the countries had some plans to evaluate the progress toward the endgame goal. However, the level of detail in the description of the evaluation plan varied. For some countries, holistic elements of the evaluation of public health policies were discussed (Norway), whereas other countries had a more detailed monitoring and evaluation plan (Scotland). Other countries described regular assessments of the effects of the measures but with less detail (Finland, Sweden).

### Are the countries progressing toward the endgame goal with the selected measures?

According to WHO, regular daily smoking rates differed between countries from 24% (France) to 7% (Norway) (Table 4, Panel C). It was estimated that the prevalence of current tobacco use will decrease from 2022 to 2025 in all the countries (Table 4, Panel A vs Panel B). For most of the included countries, the decline in tobacco use prevalence from 2022 to 2025 is estimated to be about 1–3 percentage points. E-cigarette use and smokeless tobacco use differed between countries (Table 4, Panel D, Panel E). E-cigarette use was most prevalent in the UK, Belgium, France, and Ireland, while smokeless tobacco use was the most prevalent in Norway and Sweden. Overall, it seems that current progress is not sufficient for countries to reach their endgame goals (prevalence <5%) in due time.

**Table 4:** Prevalence (%) of tobacco, smokeless tobacco and e-cigarette use in European endgame countries

	*Panel A^a^*	*Panel B^a^*	*Panel C^b^*	*Panel D^c^*	*Panel E^c^*
*Country*	*Current tobacco use prevalence 2022* *Age ≥15 y%*	*Current tobacco use prevalence estimate to 2025* *Age ≥15 y%*	*Regular daily smokers in population* *Age ≥15 y Year (%)*	*Current e-cigarette use* *Year (%), age group*	*Current smokeless tobacco use* *Year (%), age group*
Belgium	24.7	22.3	2018 (15.4)	2021 (10.0), ≥15 y	N/A
Finland	19.6	16.6^d^	2020 (12.0)	2020–2021 (2.0), ≥20 y	2020–2021 (7.0), ≥20 y
France	29.2	28.9	2019 (24.0)	2021 (6.7), 18–75 y	N/A
Ireland	18.2	16.8	2022 (18.0)	2022 (6.0), ≥15 y	N/A
The Netherlands	20.1	18.7	2021 (14.7)	2020 (1.0), ≥15 y	N/A
Norway	14.0	12.0	2022 (7.0)	N/A	2022 (18.0), 16–74 y
Slovenia	18.1	17.3	2019 (17.4)	2021 (1.3), ≥18 y	2021 (1.3), ≥18 y
Sweden	22.1	20.1^d^	2021 (9.7)	2022 (2.0), 16–84 y	2022 (14.0), 16–84 y
United Kingdom^e^	13.1	11.5	2021 (14.5)	2021 (7.7), ≥16 y	N/A

N/A: data not available.

a WHO 2024. WHO global report on trends in prevalence of tobacco use 2000–2030. Geneva: World Health Organization; 2024. Licence: CC BY-NC-SA 3.0 IGO Available from: https://www.who.int/publications/i/item/9789240088283 (accessed 31 January 2024).

b WHO European Region 2022. European Health Information Gateway. % of regular daily smokers in the population, age 15+. Available from: https://gateway.euro.who.int/en/indicators/hfa_421-3010-of-regular-daily-smokers-in-the-population-age-15plus/ (accessed 1 February 2024).

c WHO 2023. The Global Health Observatory. Most recent nationally representative survey reporting prevalence of current smokeless tobacco use or current e-cigarette use among adults (Tobacco control: Monitor). Available from: https://www.who.int/data/gho/data/indicators/indicator-details/GHO/gho-tobacco-control-monitor-survey-reporting-prevalence-of-smokeless-tobacco-use-or-e-cigarette-use-among-adults (accessed 1 February 2024).

d The estimates for the year 2025 for Finland (13.8%) and Sweden (10.8%) in the WHO (2024) report seem incorrect based on the observed prevalence for 2022 in Panel A and in the earlier WHO (2021) report. Thus, estimates for Finland and Sweden are taken from the earlier report: WHO global report on trends in prevalence of tobacco use 2000–2025, fourth edition. Geneva: World Health Organization; 2021. Licence: CC BY-NC-SA 3.0 IGO. Available from: https://www.who.int/publications-detail-redirect/9789240039322 (accessed 1 February 2024).

e Data from Scotland and England are unavailable in the WHO databases, so prevalence is presented for UK as global.

## DISCUSSION

In this article, we reviewed the current knowledge on the effectiveness of proposed tobacco endgame measures and identified future research needs. We also examined how the endgame goal has been operationalized in the national strategies among European countries with official tobacco endgame goals.

Existing scientific reviews highlighted sufficient evidence (over 5 studies) on tobacco endgame policies of VLNC, increases in tobacco tax that make tobacco products generally unaffordable, and restrictions on tobacco retailers^1,9^. An update of the literature was carried out for the tobacco-free generation (TFG) policy, which then reached sufficient evidence that was set as more than 5 studies on the topic.

The VLNC policy was the most studied tobacco endgame policy. However, research is needed to estimate its feasibility, its potential effects on the use of other tobacco products and in terms of mental and physical health, responses of the tobacco industry and illicit market to this policy measure as well as the adequate nicotine threshold of the products to alleviate nicotine craving. Although compensatory smoking might occur when smoking VLNC cigarettes, studies imply this is not the case^37,38^. Compensation might be nicotine-dose dependent and decrease when nicotine content is substantially reduced, so possible compensation could be accounted for in cigarette design^37,38^. ‘Light’ cigarettes were marketed by the tobacco industry as less harmful than non-light cigarettes due to the lower amount of tar, yet this allegation has been scientifically repelled^39^. It might be that the marketing of VLNC cigarettes by the tobacco industry could rely on similar aspects of reduced exposure to that of light cigarettes^39^. Indeed, studies imply that smokers have misperceptions considering the harmfulness of VLNC cigarettes^40,41^. Light cigarettes have been banned in the EU since 2001^42^.

The increase in tobacco taxes and the restriction of tobacco retailers resulted in effectively reducing smoking prevalence. However, there is evidence of gaps in price elasticity and feasibility of tobacco retailer restrictions in some European countries. For example, the estimates for price elasticity^43^ should be reassessed in situations where the availability of a product is heavily restricted, and this should be done for different products and by different population subgroups.

Evidence on the TFG policy reported population-level improvements but also uncertainty as to whether the objective of the targeted maximum smoking prevalence can be achieved, especially for adolescents due to their reactions to age-restrictive laws. In addition to the literature review, recent studies in the USA demonstrate that Tobacco 21 laws are widely supported and have positive results regarding the reduction of smoking in young adults and adolescents^44,45^. However, the majority of these policies have not yet been implemented, and their impact has not been assessed in European countries with an adopted endgame strategy. The final result is a lack of evidence of their wanted and unwanted impacts on this target population. Further research is needed to improve estimates of the effectiveness of endgame policies. Furthermore, studies are needed to evaluate the impact of endgame policies, not only in European populations but also in populations in the early stage of the tobacco epidemic^35^.

There remains large uncertainty on the feasibility of tobacco endgame measures. There are also differences in what measures are being considered as tobacco endgame measures in relation to new tobacco and nicotine products. For example, McDaniel et al.^1^ did not consider the implications of e-cigarettes for endgame purposes, justifying it with the challenge of assessing their role in different endgame scenarios. This is due to, for example, the controversy over these products’ marketing and use, the lack of long-term research on their health effects, variability among the products, and the political dynamics of rapid acquisition of e-cigarette companies by cigarette companies. On the other hand, Puljević et al.^9^ presented ‘moving consumers to reduced risk products’ as one endgame policy with multiple evidence syntheses (8 studies on the topic). There is no widely accepted definition of harm reduction yet in the endgame approach where the aim is to minimize the overall health harms, the harm reduction approach should reduce the risks for all the different population groups, not only for individuals in some groups (e.g. smokers)^46-48^. The actual effects of a shift from conventional cigarettes to alternative nicotine products are usually guided by behavioral tendencies that are difficult to control only with policy interventions^49^. For example, in Italy, despite the sudden increase in novel tobacco products after their introduction, the majority of smokers were still loyal to conventional cigarettes, and more than half of novel product users kept on smoking conventional cigarettes^50^. Similar results have been found elsewhere^51^. In JATC2 WP9, harm reduction policies have not been considered as tobacco endgame measures.

Ten European countries were identified as having an official endgame goal: Belgium, Finland, France, Ireland, The Netherlands, Norway, Slovenia, Sweden, England, and Scotland. Most of them had defined the goal by a specific prevalence of tobacco product use and/or objectives related to a TFG or tobacco-free society. Prevalence-related objectives were defined as a use level of 5% or less. Also, in non-European endgame countries, such as New Zealand, the objectives relate to these two measures. Similarly, one of the objectives of the European Beating Cancer Plan (‘Tobacco-free Generation’ with a prevalence of 5% or less in 2040) is in line with these objectives^2^. In some countries, the objective included, in addition to combustible tobacco use, also nicotine use. Considering the extensiveness of the endgame approach in the EU, most of the member states have yet to set an endgame goal overall. So, the countries included could be seen at the forefront of tobacco control, with the aim of ending the tobacco epidemic^1^.

According to the assessment of the national endgame policy documents, all included countries were rather similar considering the adopted measures. For example, all countries had adopted some product standards in their endgame measures, mainly bans on characterizing flavors and additives. These bans were usually based on the EU TPD^36^. A more detailed list of allowed ingredients in products could be considered as manufacturers have made efforts to circumvent the ban^52^. One recent example is given by the Netherlands’ restrictive list of flavoring additives in e-liquids^53^. Tax increases were another commonly adopted measure in the endgame countries, yet adopted increases were modest in terms of a definition of an endgame measure that would be to increase them to make tobacco products generally unaffordable. Strategies to reduce tobacco availability, for example, by licensing systems and/or banning sales in some places, were adopted in all included countries. Examples of banning sales in certain places, such as in supermarkets and gas stations in the Netherlands and France, would give valuable information on possible challenges for other countries to acknowledge when planning to implement such a measure. Notably, the measure with the most studies on VLNC was acknowledged only in France among the European endgame countries. The measures the countries had adopted included, based on an earlier classification^9^, all but institutional-focused measures. Additionally, national policy documents lacked considerations of future technological and cultural changes in societies and their possible effects on the endgame goal or adapted measures beyond discussion on new tobacco and nicotine products already on the market.

Overall, important evidence gaps remain on the feasibility of different endgame measures for European countries. Most of the prior evidence comes from other countries, such as the USA, and more research on the possible effects in European countries is warranted. It must be noted that part of the adaptation of regulations is based on the implementation of the EU TPD^36^. It seems TPD is successful in setting some standards on regulations for the member states, such as those relating to product standards (such as the menthol ban). However, presently, TPD is less capable of supporting countries adopting and implementing more advanced measures than required by the directive. The procedure of notification to the Commission about national measures is one example that might hinder the adaptation of advanced endgame measures at the national level.

All the reviewed endgame policy documents included some mentions of vulnerable groups. These mentions were related to smoking cessation as well as to other measures. Identified vulnerable groups were most commonly adolescents but also pregnant women and persons with low socio-economic position or mental health problems. Certain vulnerable groups were, however, neglected by most documents (e.g. LGBTQ+). Disappointedly, practical actions related to vulnerable groups were uncommon, as only a few countries planned measures to reduce tobacco use, especially among these groups. Pro-equity measures should be emphasized when planning, adopting, and implementing future tobacco control measures to account for different population groups, especially those most vulnerable^54^. Smoking cessation measures were given strong emphasis in country strategies, but incorporating cessation measures in specific endgame measures was lacking. These measures could be linked to, for example, VLNC and high tax increases. In general, cessation support is one of the domains where European countries need to improve the implementation of measures laid down in Article 14 of the WHO FCTC^55^.

Countries had different plans for evaluating the progress toward the endgame objective. Some countries had a comprehensive evaluation plan, such as Scotland, while some reported more general activities. Based on WHO prevalence estimates, the current progress in decreasing tobacco use is not sufficient for countries to reach their endgame goals, especially countries with an earlier endgame goal (e.g. Ireland in 2025). Recent trends in tobacco and nicotine product market share and use patterns propose a change from exclusive cigarette use to multiple product use^56,57^. These trends could influence meeting the objectives of an endgame goal. For example, observed increases in snus use seem to impede meeting the goal of a tobacco- and nicotine-free Finland^58^. More comprehensive measures should be adopted and implemented also for other countries to meet the objectives based on the historical trends of use. Furthermore, publishing estimates of the future prevalence of different tobacco and nicotine product use by the WHO would be beneficial for countries incorporating them into their endgame goals.

There are many countries that have adopted different tobacco control measures but do not have an official endgame objective, such as Iceland, Hungary, and Ukraine. NGOs in different countries, such as Greece, Germany, Romania, and Spain, have created endgame goals or proposals, but they are not objectives of the government^59^. Supporting these and other countries that have not yet presented endgame goals, to adopt a national endgame objective would provide synergies for European countries in preventing tobacco use and accomplishing national and international tobacco endgame aims.

### Strengths and limitations

Our investigation concentrated on European countries with a national endgame goal. Future studies should investigate other regions additionally. Mentions of different policy measures in each country’s policy document(s) should be viewed carefully in terms of actual changes in legislation; mentioning these policies do not guarantee their further enactment and implementation. Some of the measures have been implemented after the publication of the strategies, such as plain packaging and bans on characterizing flavors in several countries. Estimating the detailed effects, such as point estimates, of the adopted policy measures in national and in European contexts, or which model of tobacco endgame is the most effective, was beyond the scope of this study. Nevertheless, our review gives a comprehensive description of the European endgame countries and their adopted strategies at one study point, providing detailed information for cross-country comparisons and further developing and assessing the tobacco endgame situation in Europe and elsewhere.

## CONCLUSION

This review identified ten European countries that have adopted an official endgame goal, which usually considers a prevalence objective of use of no more than 5% by a certain year. Based on the current prevalence estimates of tobacco use, achieving the goals will be challenging unless more emphasis and specific measures are implemented to curb tobacco and nicotine product use. In some of the countries with the endgame goals, WHO FCTC and MPOWER measures have not yet been adopted fully, which also can be seen as a barrier to achieving the goals. In addition to implementing these measures, new innovative strategies and measures to target the objective of an endgame would also be essential. The current evidence on these strategies and measures is still limited and further studies are necessary, but their positive impact has been suggested.

## Supplementary Material



## Data Availability

The data supporting this research are available from the authors on reasonable request.

## References

[1] McDaniel PA, Smith EA, Malone RE. The tobacco endgame: a qualitative review and synthesis. Tob Control. 2016;25(5):594-604. doi:10.1136/tobaccocontrol-2015-052356 26320149 PMC5036259

[2] European Commission. Europe’s Beating Cancer Plan Communication from the commission to the European Parliament and the Council. European Commission; 2022. Accessed May 23, 2023. https://health.ec.europa.eu/system/files/2022-02/eu_cancer-plan_en_0.pdf

[3] European Commission. Attitudes of Europeans towards tobacco and electronic cigarettes. Special Eurobarometer 506. European Commission; 2021. Accessed January 12, 2024. https://europa.eu/eurobarometer/surveys/detail/2240

[4] European Commission. Europe’s Beating Cancer Plan: Commission publishes implementation roadmap. European Commission; 2021. Accessed May 22, 2023. https://ec.europa.eu/newsroom/sante/items/727246/en

[5] Straarup MS, O’Donovan F, Lambrou A, et al. The Joint Action on Tobacco Control: a cooperation project for strengthening tobacco control in Europe. Tob Prev Cessat. 2022;8:26. doi:10.18332/tpc/151050 35855292 PMC9251630

[6] JATC2 WP9 partners. M9.1 Indicator compendium - WP9 “Best practices to develop an effective and comprehensive tobacco endgame strategy.” Joint Action on Tobacco Control 2. Outcomes and useful material. 2023. www.jaotc.eu

[7] Edwards R, Hoek J, Karreman N, et al. Evaluating tobacco industry ‘transformation’: a proposed rubric and analysis. Tob Control. 2022;31(2):313–321. doi:10.1136/tobaccocontrol-2021-056687 35241605

[8] Greenhalgh T, Thorne S, Malterud K. Time to challenge the spurious hierarchy of systematic over narrative reviews?. Eur J Clin Invest. 2018; 48:e12931. 10.1111/eci.12931 29578574 PMC6001568

[9] Puljević C, Morphett K, Hefler M, et al. Closing the gaps in tobacco endgame evidence: a scoping review. Tob Control. 2022;31(2):365-375. doi:10.1136/tobaccocontrol-2021-056579 35241614

[10] Cornell University Library. A guide to evidence synthesis: types of evidence synthesis. Ithaca, NY: Cornell University Library, 2021.

[11] Scollo M, Bayly M. 13.3 The price of tobacco products in Australia. In Greenhalgh, EM, Scollo, MM and Winstanley, MH [editors]. Tobacco in Australia: Facts and issues. Melbourne: Cancer Council Victoria; 2023. Accessed February 1, 2024. http://www.tobaccoinaustralia.org.au/chapter-13-taxation/13-3-the-price-of-tobacco-products-in-australia

[12] Tait P, Saunders C, Rutherford P, Tobacco Control Research Turanga, Lincoln University (Canterbury, N.Z.). Agribusiness and Economics Research Unit. Quota Management Policy for New Zealand Tobacco Supply. Tobacco Control Research Turanga, School of Population Health, University of Auckland; 2013. Accessed April 12, 2024. http://www.turanga.org.nz/sites/turanga.org.nz/files/Quota%20Policy%20Report%20online.pdf

[13] van der Deen FS, Wilson N, Cleghorn CL, et al. Impact of five tobacco endgame strategies on future smoking prevalence, population health and health system costs: two modelling studies to inform the tobacco endgame. Tob Control. 2018;27(3):278-286. doi:10.1136/tobaccocontrol-2016-053585 28647728

[14] McNeill A, Guignard R, Beck F, et al. Understanding increases in smoking prevalence: case study from France in comparison with England 2000-2010. Addiction 2015110(3):392-400. doi:10.1111/add.12789 25393099

[15] Tiessen J, Hunt P, Celia C, et al. Assessing the impacts of Revising the Tobacco Products Directive. Study to support a DG SANCO Impact Assessment. RAND Corporation, 2011. Accessed August 15, 2023. https://www.rand.org/pubs/technical_reports/TR823.html 10.7249/rhq-01-01-13 PMC4945220 28083169

[16] Ait Ouakrim D, Wilson T, Waa A, et al. Tobacco endgame intervention impacts on health gains and Māori:non-Māori health inequity: a simulation study of the Aotearoa/New Zealand Tobacco Action Plan. Tob Control. Published online first 10 January 2023. doi:10.1136/tc-2022-057655 PMC11672025 36627213

[17] de Leon K, Sarita JT. The Philippines: pioneering the tobacco endgame. Tob Control. 13 January 2020. Accessed January 8, 2024. https://blogs.bmj.com/tc/2020/01/13/the-philippines-pioneering-the-tobacco-endgame/

[18] Dyer O. New Zealand plans to outlaw tobacco sales to citizens born after 2008. BMJ. 2021; 375:n3057. doi:10.1136/bmj.n3057 34893520

[19] Walters EH, Barnsley K. Tobacco-free generation legislation. Med J Aust. 2015;202(10):509510. doi:10.5694/mja15.00416 26021352

[20] Trainer E, Gall S, Smith A, et al. Public perceptions of the tobacco-free generation in Tasmania: adults and adolescents. Tob Control. 2017;26(4):458-460. doi:10.1136/tobaccocontrol-2016-053105 27466060

[21] Berrick J. Worldwide news and comment: US: Brookline introduces Tobacco-Free Generation law. Tobacco Control. 2022;31:399-401. doi:10.1136/tobaccocontrol-2022-057419

[22] Fernández Megina R, Radu-Loghin C, Rodriguez Lozano F. European Citizens’ Initiative “Call to achieve a tobacco-free environment and the first European Tobacco-Free Generation by 2030”. Tob Prev Cessation. 2023;9:A121. doi:10.18332/tpc/162826

[23] Linnansaari A, Ollila H, Pisinger C, et al. Towards Tobacco-Free Generation: implementation of preventive tobacco policies in the Nordic countries. Scand J Public Health. 2023;51(8):1108-1121. doi:10.1177/14034948221106867 35799463 PMC10642214

[24] Willemsen MC, Been JV. Accelerating tobacco control at the national level with the Smoke-free Generation movement in the Netherlands. NPJ Prim Care Respir Med. 2022;32(1):58. doi:10.1038/s41533-022-00321-8 36564383 PMC9780621

[25] Been JV, Laverty AA, Tsampi A, et al. European progress in working towards a tobacco-free generation. Eur J Pediatr. 2021;180(12):3423-3431. doi:10.1007/s00431-021-04116-w 34032890 PMC8589739

[26] Hefler M, Bianco E, Bradbrook S, et al. What facilitates policy audacity in tobacco control? An analysis of approaches and supportive factors for innovation in seven countries. Tob Control. 2022;31(2):328-334. doi:10.1136/tobaccocontrol-2021-056570 35241607

[27] Johannesen CK, Andersen S, Bast LS. Estimating future smoking in Danish youth - effects of three prevention strategies. Scand J Public Health. 2021;49(8):931-939. doi:10.1177/1403494820942678 32791944

[28] Berrick J. Drawing on Adolescent psychology to achieve tobacco-free generations. Public Health Rev. 2022;43:1604321. doi:10.3389/phrs.2022.1604321 36211226 PMC9533236

[29] Nuyts PAW, Kuijpers TG, Willemsen MC, et al. How can a ban on tobacco sales to minors be effective in changing smoking behaviour among youth? A realist review. Prev Med. 2018;115:61-67. doi:10.1016/j.ypmed.2018.08.013 30144483

[30] Berrick J, Gkritza K. Adolescent noncompliance with age-specific versus universal US motorcycle helmet laws: systematic review and meta-analysis. J Saf Res. 2021;76:166–175. doi:10.1016/j.jsr.2020.12.011 33653548

[31] Friedman AS, Buckell J, Sindelar JL. Tobacco-21 laws and young adult smoking: quasi-experimental evidence. Addiction. 2019;114(10):1816-1823. doi:10.1111/add.14653 31342591 PMC7233410

[32] Friedman AS, Wu RJ, Do local tobacco-21 laws reduce smoking among 18 to 20 year-olds?, Nicotine Tob Res. 2020;22(7):1195-1201.doi:10.1093/ntr/ntz123 31348515

[33] Nuyts PAW, Kuipers MAG, Willemsen MC, et al. An increase in the tobacco age-of-sale to 21: for debate in Europe. Nicotine Tob Res. 2020;22(7):1247-1249. doi:10.1093/ntr/ntz135 31408159 PMC7291794

[34] Bryan C, Hansen B, McNichols D, et al. Do state Tobacco 21 Laws work? NBER Working Paper Series, Working paper 28173. National Bureau of Economic Research, 2021. Accessed January 8, 2024. https://www.nber.org/system/files/working_papers/w28173/w28173.pdf

[35] Thun M, Peto R, Boreham J, et al. Stages of the cigarette epidemic on entering its second century. Tob Control. 2012;21(2):96-101. doi:10.1136/tobaccocontrol-2011-050294 22345230

[36] Directive 2014/40/EU of the European Parliament and of the Council of 3 April 2014 on the approximation of the laws, regulations and administrative provisions of the Member States concerning the manufacture, presentation and sale of tobacco and related products and repealing Directive 2001/37/EC Text with EEA relevance. Eur-Lex. Accessed 18 March 2024. https://eur-lex.europa.eu/eli/dir/2014/40/oj

[37] Donny EC, White CM. A review of the evidence on cigarettes with reduced addictiveness potential. Int J Drug Policy. 2022;99:103436. doi:10.1016/j.drugpo.2021.103436 34535366 PMC8785120

[38] Benowitz NL, Henningfield JE. Reducing the nicotine content to make cigarettes less addictive. Tob Control. 2013;22(1):i14–17. doi:10.1136/tobaccocontrol-2012-050860 23591498 PMC3632983

[39] Pederson LL, Nelson DE. Literature review and summary of perceptions, attitudes, beliefs, and marketing of potentially reduced exposure products: communication implications. Nicotine Tob Res. 2007;9(5):525-534. doi:10.1080/14622200701239548 17454709

[40] Johnson AC, Mercincavage M, Souprountchouk V, et al. Responses to reduced nicotine cigarette marketing features: a systematic review. Tob Control. 2023;32:366-374. doi:10.1136/tobaccocontrol-2021-056826 PMC8986886 34620718

[41] Byron MJ, Jeong M, Abrams DB, et al. Public misperception that very low nicotine cigarettes are less carcinogenic. Tob Control. 2018;27(6):712-714. doi:10.1136/tobaccocontrol-2017-054124 29363610 PMC6056339

[42] Directive 2001/37/EC of the European Parliament and of the Council of 5 June 2001 on the approximation of the laws, regulations and administrative provisions of the Member States concerning the manufacture, presentation and sale of tobacco products - Commission statement. Eur-Lex. Accessed March 18, 2024. https://eur-lex.europa.eu/legal-content/en/ALL/?uri=CELEX%3A32001L0037

[43] Zimring FE, Nelson W. Cigarette taxes as cigarette policy. Tob Control. 1995;4(1):S25–35. doi:10.1136/tc.4.suppl1.S25

[44] Colston DC, Xie Y, Thrasher JF, et al. Tobacco 21 laws may reduce smoking and tobacco-related health disparities among youth in the U.S. Prev Med Rep. 2022;27:101762. doi:10.1016/j.pmedr.2022.101762 35340271 PMC8943436

[45] Morain SR, Garson A, Raphael JL. State-level support for tobacco 21 laws: results of a Five-State Survey. Nicotine Tob Res. 2018;20(11):1407-1411. doi:10.1093/ntr/ntx208 29059407

[46] Marlatt GA. Harm reduction: come as you are. Addict Behav. 1996;21(6):779-788. doi:10.1016/0306-4603(96)00042-1 8904943

[47] Beirness DJ, Jesseman R, Notarandrea R, et al. Harm reduction: what’s in a name? Canadian Centre on Substance Use and Addiction, Ottawa, CCSA, May 2008. Accessed January 8, 2024. https://www.ccsa.ca/harm-reduction-whats-name

[48] Leone FT, Carlsen K-H, Chooljian D, et al. Recommendations for the appropriate structure, communication, and investigation of tobacco harm reduction claims. An official American Thoracic Society Policy Statement. Am J Resp Crit Care. 2018;198(8):e90-e105. doi:10.1164/rccm.201808-1443ST PMC6943880 30320525

[49] Gallus S, Stival C, McKee M, et al. Impact of electronic cigarette and heated tobacco product on conventional smoking: an Italian prospective cohort study conducted during the COVID-19 pandemic. Tob Control. 2024;33(2):267-270. doi:10.1136/tc-2022-057368 36207129

[50] Carreras G, Minardi V, Lugo A, et al. Italians are still loyal to conventional cigarettes, despite novel tobacco products. Ann Ist Super Sanita. 2022;58(4):264-268. doi:10.4415/ANN_22_04_06 36511197

[51] Pasquereau A, Quatremère G, Guignard R, et al. Usage de la cigarette électronique, tabagisme et opinions des 18-75 ans. Baromètre de Santé publique France 2017. Saint-Maurice: Santé publique France; 2019.

[52] Brink A, Glahn AS, Kjaer NT. Tobacco companies’ exploitation of loopholes in the EU ban on menthol cigarettes: a case study from Denmark. Tob Control. 2023;32(6):809-812. doi:10.1136/tobaccocontrol-2021-057213 35314507 PMC10646948

[53] Pennings JLA, Havermans A, Krüsemann EJZ, et al. Reducing attractiveness of e-liquids: proposal for a restrictive list of tobacco-related flavourings. Tob Control. 2024;33(e1):e41-e47. doi:10.1136/tc-2022-057764 36669881 PMC10958261

[54] Mills SD, Rosario C, Yerger VB, Kalb MD, Ribisl KM. Recommendations to advance equity in tobacco control. Tob Control. Published online first 19 December 2022. doi:10.1136/tc-2022-057670 PMC10277310 36535756

[55] González-Marrón A, Koprivnikar H, Tisza J, et al. Tobacco endgame in the WHO European Region: feasibility in light of current tobacco control status. Tob Induc Dis. 2023;21:1–16. doi:10.18332/tid/174360 PMC10647070 38026503

[56] Liu Y, Filippidis FT. Tobacco market trends in 97 countries between 2007 and 2021. Tob Induc Dis. 2024;22:31. doi:10.18332/tid/177441 PMC10835573 38314375

[57] Chen DTH, Girvalaki C, Mechili Enkeleint A, et al. Global patterns and prevalence of dual and poly-tobacco use: a systematic review. Nicotine Tob Res. 2021;23(11):1816–1820. doi:10.1093/ntr/ntab084 34009377 PMC8825763

[58] Ruokolainen O, Reinikainen J, Ollila H, Härkänen T. Will the objective of a tobacco and nicotine free Finland by 2030 be achieved? A modelling study among the Finnish adult population. Yhteiskuntapolitiikka. 2023;88(5–6):500-512. https://urn.fi/URN:NBN:fi-fe20231212153359

[59] Garen L, Schaller K. Strategy for a tobacco-free Germany 2040. German Cancer Research Center, 2021. Accessed January 8, 2024. https://unfairtobacco.org/wp-content/uploads/2022/05/2021_Strategy-for-a-tobacco-free-Germany-2040.pdf

