# Supplementary material for: Tobacco endgame measures and their adaptation in selected European countries: A narrative review synthesis
Source: Tob Prev Cessat. 2024 Apr 18;10:10.18332/tpc/186402. doi: 10.18332/tpc/186402 (PMC11025294; doi:10.18332/tpc/186402)
Supplement: Supplementary file 1 [file TPC-10-18-s1.pdf]

Supplementary Material for 'Tobacco endgame measures and their adaptation in selected European countries: a review synthesis'

Supplementary Material

Appendix A. String for the update on tobacco-free generation

(smoking[Title] OR smoker*[Title] OR tobacco[Title] OR cigarette*[Title] OR nicotine[Title])
AND
((review*[TW] OR consensus[TW] OR commentary[TW] OR meta-analysis[TW] OR synthesis[TW] OR "expert opinion"[TW] OR modelling[TW] OR modeling[TW]) OR (dynamic*[TW] OR "life table" [TW] OR forecast*[TW] OR simulation[TW] AND model[TW]))
AND
("tobacco free generation*"[TW] OR "tobacco-free generation*"[TW] OR "age-ofsale"[TW] OR "age of sale"[TW] OR "smoke-free generation*"[TW] OR "smoke free generation*"[TW])
AND
("2021/03/01"[Date - Publication] : "3000"[Date - Publication])

Appendix B. Country policy documents

Belgium:

Sante Publique, Securite de la Chaine Alimentaire et Environnement. Stratégie interfédérale 2022-2028 pour une génération sans tabac, 2022. (Available: https://organesdeconcertation.sante.belgique.be/fr/documents/strategie-interfederale-2022-2028-pour-une-generation-sans-tabac)

Finland:

Roadmap to a tobacco-free Finland: action plan on tobacco control. Publications of the Ministry of Social Affairs and Health 12. Ministry of Social Affairs and Health, 2014. (Available: http://urn.fi/URN:ISBN:978-952-00-3513-6)

Development of tobacco and nicotine policy: proposals for action by the working group 2023. Reports and memorandums 14. Helsinki: Ministry of Social Affairs and Health, 2023. (Available: http://urn.fi/URN:ISBN:978-952-00-8353-3)

France:

Programme national de lutte contre le tabac 2023–2027. Édition: Ministère de la santé et de la prévention. Direction générale de la santé conception & maquettage : dicom des ministères sociaux / parimage. Novembre 2023. (Available: https://sante.gouv.fr/IMG/pdf/programme_national_contre_le_tabac.pdf)

Ireland:

Tobacco Free Ireland. Report of the Tobacco Policy Review Group. Department of Health, 2013 (Available: https://assets.gov.ie/19465/0c99a96e05c54b249c7d53b93b17437c.pdf)

Department of Health. Tobacco-free Ireland Action Plan. Department of Health, 2013 (Available: https://assets.gov.ie/15942/ebab88a03d8f42bca3498db35f900125.pdf)

Netherlands:

The National Prevention Agreement. A healthier Netherlands. Ministry of Health, Welfare and Sport, 2019 (Available: https://www.government.nl/topics/smoking/documents/reports/2019/06/30/the-national-prevention-agreement)

Norway:

Folkehelsemeldinga. Nasjonal strategi for utjamning av sosiale helseforskjellar. Det Kongelege Helse- og Omsorgsdepartement, 2023. (Available: https://www.regjeringen.no/no/dokumenter/meld.-st.-15-20222023/id2969572/)

Folkehelsemeldinga. Gode liv i eit trygt samfunn. Det Kongelege Helse- og Omsorgsdepartement, 2019. (Available: https://www.regjeringen.no/contentassets/84138eb559e94660bb84158f2e62a77d/nn-no/pdfs/stm201820190019000dddpdfs.pdf)

Public Health Report – A Good Life in a Safe Society (short version). White Paper No. 19 (2018-2019). Ministry of Health and Care Services, 2020. (Available: https://www.regjeringen.no/contentassets/84138eb559e94660bb84158f2e62a77d/nn-no/sved/publichealthreport.pdf)

Slovenia:

Za Slovenijo Brez tobaka 2022-2030. Strategija za zmanjševanje posledic rabe tobaka. Ljubljana: Vlada Republike Slovenije, 2022 (Available: https://www.gov.si/assets/ministrstva/MZ/DOKUMENTI/ZDRAVJE/Preventiva-in-skrb-za-zdravje/Strategija-za-Slovenijo-brez-tobaka.pdf)

Towards tobacco-free Slovenia 2040. Ljubljana: National Institute of Public Health (NIJZ), 2021 (Available: https://nijz.si/wp-content/uploads/2022/02/towards_tobacco-free_slovenia_2040.pdf)

Sweden:

En samlad strategi för alkohol-, narkotika-, dopnings- och tobakspolitiken 2016–2020. Regeringens skrivelse 2015/16:86. Stockholm: Socialdepartementet, 2016 (Available: https://www.regeringen.se/rattsliga-dokument/skrivelse/2016/02/skr.20151686)

En samlad strategi för alkohol-, narkotika-, dopnings- och tobakspolitiken samt spel om pengar 2022–2025. Regeringens skrivelse 2021/22:213. Stockholm: Socialdepartementet, 2022 (Available: https://www.regeringen.se/rattsliga-dokument/skrivelse/2022/03/skr.-202122213)

UK, England:

The smokefree 2030 ambition for England. Research briefing by Bukky Balogun, 16 October 2023. House of Commons Library. (Available at: https://commonslibrary.parliament.uk/research-briefings/cbp-9655/)

Towards a Smokefree Generation: A Tobacco Control Plan for England. Department of Health, 2017 (Available: https://assets.publishing.service.gov.uk/government/uploads/system/uploads/attachment_data/file/630217/Towards_a_Smoke_free_Generation_-_A_Tobacco_Control_Plan_for_England_2017-2022__2_.pdf)

Advancing our health: prevention in the 2020s. HM Government, 2019 (Available: https://assets.publishing.service.gov.uk/government/uploads/system/uploads/attachment_data/file/819766/advancing-our-health-prevention-in-the-2020s-accessible.pdf)

UK, Scotland:

Tobacco and vaping framework: roadmap to 2034. Scottish Government, November 2023. (Available: https://www.gov.scot/publications/tobacco-vaping-framework-roadmap-2034/pages/11/#page-top=)

Creating a Tobacco-Free Generation. A Tobacco Control Strategy for Scotland. Edinburgh: The Scottish Government, 2013 (Available: https://www.gov.scot/binaries/content/documents/govscot/publications/strategy-plan/2013/03/tobacco-control-strategy-creating-tobacco-free-generation/documents/creating-tobacco-free-generation-tobacco-control-strategy-scotland/creating-tobacco-free-generation-tobacco-control-strategy-scotland/govscot%3Adocument/00417331.pdf)

© 2024 Ruokolainen O. et al.
